# Effect of sous-vide cooking conditions on the physicochemical, microbiological and microstructural properties of duck breast meat

**DOI:** 10.5713/ab.23.0039

**Published:** 2023-06-26

**Authors:** Dong-Min Shin, Jong Hyeok Yune, Dong-Hyun Kim, Sung Gu Han

**Affiliations:** 1 Department of Food Science and Biotechnology of Animal Resources, Konkuk University, Seoul 05029, Korea

**Keywords:** Duck, Meat Quality, Microbial Safety, Microstructure, Sous-vide, Texture

## Abstract

**Objective:**

Sous-vide cooking offers several advantages for poultry meat, including enhanced tenderness, reduced cooking loss, and improved product yield. However, in duck meat, there are challenges associated with using the sous-vide method. The prolonged cooking time at low temperatures can lead to unstable microbial and oxidative stabilities. Thus, we aimed to assess how varying sous-vide cooking temperatures and durations affect the physicochemical and microbial characteristics of duck breast meat, with the goal of identifying an optimal cooking condition.

**Methods:**

Duck breast meat (*Anas platyrhynchos*) aged 42 days and with an average weight of 1,400±50 g, underwent cooking under various conditions (ranging from 50°C to 80°C) for either 60 or 180 min. Then, physicochemical, microbial, and microstructural properties of the cooked duck breast meat were assessed.

**Results:**

Different cooking conditions affected the quality attributes of the meat. The cooking loss, lightness, yellowness, Hue angle, whiteness, and thiobarbituric acid reactive substance (TBARS) values of the duck breast meat increased with the increase in cooking temperature and time. In contrast, the redness and chroma values decreased with the increase in cooking temperature and time. Cooking of samples higher than 60°C increased the volatile basic nitrogen contents and TBARS. Microbial analysis revealed the presence of *Escherichia coli* and Coliform only in the samples cooked at 50°C and raw meat. Cooking at lower temperature and shorter time increased the tenderness of the meat. Microstructure analysis showed that the contraction of myofibrils and meat density increased upon increasing the cooking temperature and time.

**Conclusion:**

Our data indicate that the optimal sous-vide method for duck breast meat was cooking at 60°C for 60 min. This temperature and time conditions showed good texture properties and microbial stability, and low level of TBARS of the duck breast meat.

## INTRODUCTION

Meats require heat treatment to improve palatability and digestive efficiency, and especially to delay microbial growth. Meat proteins undergo modifications depending on the cooking temperature and time, which affect the quality attributes of the meat product, including cooking loss, color, and texture [1]. Meat products can be cooked using various methods of heat transfer, such as convection, conduction, and radiation [2]. Sous-vide is a popular cooking method in which heat is transferred from the water to vacuum-sealed food through conduction [3]. In this cooking method, the vacuum-packed meat is placed in a plastic bag and cooked under controlled temperature for a specific time in a water bath [3]. The meat is then immediately cooled in an ice bath to bypass the optimal growth temperature of microbes [4]. The advantages of sous-vide include uniform heating of the meat, decreased risk of microbial recontamination after heating and during storage—as the meat is vacuum-packaged—increased meat tenderness as the meat is subjected to low heat treatment (50°C to 65°C) for a prolonged period. Moreover, cooking at low temperatures for a long time has economic benefits as it can reduce cooking loss and increase the meat product yield. Several studies have demonstrated that the sous-vide cooking method improves the overall meat quality indices, such as cooking loss, tenderness, and texture [5,6].

Duck meat, known for its unique sensory properties such as color and flavor, is produced and consumed globally, particularly in Asia. Duck meat is classified as red meat due to its higher red muscle fiber content compared to chicken or turkey meat [7]. Additionally, compared to other meats, duck meat has higher levels of unsaturated fatty acids, such as linoleic acid, linolenic acid, and oleic acid [8]. Some studies have investigated the positive health effects of these fatty acid profiles, including anti-obesity effects through the promotion of lipid metabolism in viscera tissues, and hepatic health effects by inhibiting lipotoxicity in HepG2 cells [9,10]. Thus, nutrient-rich duck meat can be a good dietary choice.

Although the sous-vide method has the aforementioned benefit in meat processing, there is limited scientific information that specifically applies to duck meat. Duck meat, when cooked using the sous-vide method which involves cooking at low temperatures for a prolonged period, is susceptible to lipid oxidation and microbial contamination due to its high content of unsaturated fatty acids. Therefore, it may be beneficial to explore optimal sous-vide cooking methods for duck meat. In this study, we investigated the effects of different cooking temperatures (50°C, 60°C, 70°C, and 80°C) and time (60 and 180 min) on the physicochemical and microbial properties of sous-vide cooked duck breast meat.

## MATERIALS AND METHODS

### Experimental design

Pekin ducks (*Anas platyrhynchos*) aged 42 days with a bodyweight of 1,400±50 g were slaughtered at a local abattoir. The skin, fat and connective tissues were removed from the breast muscles. Next, the breast muscles were vacuum-packaged in an 80 μm polyethylene vacuum pouch, stored at 4°C, and analyzed within two days. The samples were cooked at 50°C, 60°C, 70°C, or 80°C with cooking time of 60 and 180 min for each temperature. Vacuum-packed duck breasts were heated using a circulating thermostatic water bath (VS-1205SW1-0; Vision Science, Daegu, Korea). All samples were chilled in an ice bath after heating to pass the optimum temperature for microbial growth. Cooked duck breast sample stored at 4°C until further analysis.

### Cooking loss

Pre-weighted duck breast meat was vacuum-packed before cooking. Exudative liquid from cooked duck breast samples was removed, and the samples were chilled until approximately 16°C. Chilled and dried samples were weighted 10 times per treatment. Cooking loss was calculated by comparing the meat weight before and after sous-vide cooking, as follows:


$$\text{Cooking loss }(\%)=[({\text{W}}_{0}-{\text{W}}_{1})/{\text{W}}_{0}]\times 100$$

where W_0_ is the weight of raw meat batter (g), and W_1_ is weight of cooked meat batter (g).

### Instrumental color

The color of the duck breast meat was measured on the cutting side of the cooked duck breast using a colorimeter (Minolta Chroma Meter CR-210; Minolta, Tokyo, Japan; Illuminate C, calibrated with a white plate; Y, 93.5; x, 0.3134; y, 0.3197). The color wass described in terms of L* (lightness), a* (redness), b* (yellowness), ΔE (color difference), C* (chroma), H° (hue angle), and whiteness. The ΔE, C*, H°, and whiteness values were calculated using the following equations:


$$\begin{array}{l} \Delta \text{E}={\left[{\left({\text{L}}^{★}-{{\text{L}}_{0}}^{★}\right)}^{2}+{\left({\text{a}}^{★}-{{\text{a}}_{0}}^{★}\right)}^{2}+{\left({\text{b}}^{★}-{{\text{b}}_{0}}^{★}\right)}^{2}\right]}^{1/2}\\ {\text{C}}^{★}={\left[{\left({\text{a}}^{★}\right)}^{2}+{\left({\text{b}}^{★}\right)}^{2}\right]}^{1/2}\\ \text{H}{}^{\circ}=\text{tan }({\text{b}}^{★}/{\text{a}}^{★})\\ \text{Whiteness:}\;100-{\left[{\left(100-\text{L}\right)}^{2}+{\text{a}}^{2}+{\text{b}}^{2}\right]}^{1/2} \end{array}$$

### Thiobarbituric acid reactive substance value

Lipid oxidation was evaluated using the 2-thiobarbituric acid (TBA) method, following the protocols of a previous study with minor modifications [11]. The meat sample (10 g) was homogenized with 50 mL of distilled water at 10,000 rpm for 2 min using a homogenizer (Model AM-7; Nihonseiki Kaisha Ltd., Osaka, Japan). The homogenizer cup and blade were washed with 47.5 mL of distilled water, and the total mixture was transferred to a distillation tube. Next, 2.5 mL of 4 N HCl and 1 mL of antifoam agent (KMK-73; Shin-Etsu Silicone Co., LTD., Seoul, Korea) were added to the mixture in the distillation tube. The mixture was subjected to distillation and 40 mL of the distillate was collected. An aliquot of the distillate (5 mL) was added to a test tube containing 5 mL of 0.02 M TBA in 90% acetic acid (TBA reagent) and mixed using a vortex mixer (Vortex-Genie 2, Bohemia, NY, USA). The tube was sealed with a cap and heated in a water bath at 95°C for 35 min. The samples were then chilled in an ice bath for 10 min. The absorbance of the sample was measured at 538 nm using a UV/VIS spectrophotometer (Optizen 2120 UV Plus; Mecasys Co., Ltd., Daejeon, Korea). The TBA values were expressed as malonaldehyde (MDA) equivalent (mg MDA/kg of sample).

### Volatile basic nitrogen test

Protein deterioration in the duck breast meat sample was assessed using the volatile basic nitrogen (VBN) method. The meat sample (5 g) in a conical tube (SPL Life Sciences Co., Ltd., Gyeonggi, Korea) was homogenized with 15 mL distilled water at 10,000 rpm for 2 min using a homogenizer (HG-15A; DAIHAN, Wonju, Korea). Next, 30 mL of distilled water was added to the homogenate and mixed using a vortex mixer. The mixture was filtered through a Whatman filter paper No. 1 (Whatman International, Maidstone, UK). The filtrate (1 mL) and 50% K_2_CO_3_ were added to the outer section of the Conway micro diffusion cell, while 1 mL of 0.01 N H_3_BO_3_ solution and 100 μL of indicator (0.066% methyl red and 0.066% bromocresol green in ethanol) were added to the inner section. The samples were incubated at 37°C for 2 h. Finally, the solution in the inner section was titrated against 0.02 N H_2_SO_4_. The VBN content was calculated using the following equation:


VBN (mg %)=[(a-b)×(f×0.02×14.007×100×100)]/S

where *S* is the sample weight (mg), *a* is the volume (mL) of H_2_SO_4_ added to the solution obtained from the inner section, *b* is the volume (mL) of H_2_SO_4_ added to the blank, and *f* is the standard factor of H_2_SO_4_.

### Warner-Bratzler shear force measurement

The tenderness of duck breast meat was measured based on Warner-Bratzler shear force (WBSF) at room temperature using a texture analyzer (TA-XT2i, Stable Micro Systems Ltd., Godalming, UK). The core of the cooked duck breast was obtained using a hand-held coring device (1.3 cm diameter) along with the muscle fiber. A Warner-Bratzler blade with a triangular cut-out notch was used to measure the shear force. The settings of the texture analyzer were as follows: pre-test speed, 2.0 mm/s; post-test speed, 5.0 mm/s; maximum load, 2.0 kg; head speed, 2.0 mm/s; distance, 8.0 mm; force, 5.0 g. The texture analyzer was calibrated to 5 kg before use.

### Texture profile analysis

Texture profile analysis (TPA) was performed using a texture analyzer (TA-XT2i; Stable Micro Systems Ltd., England) equipped with a 45° conical probe at room temperature. The central part of the cooked duck breast was cut into a cube with a dimension of 2×2×2 cm. The settings of the texture analyzer were as follows: pre-test speed, 2.0 mm/s; post-test speed, 5.0 mm/s; maximum load, 2.0 kg; head speed, 2.0 mm/s; distance, 8.0 mm; force, 5.0 g. The texture analyzer was calibrated with 5 kg before use.

### Microbiological analysis

Microbiological analysis was conducted after the samples were cooked and chilled. The duck breast sample (25 g) was transferred to a sterilized filter bag for homogenization (ELMEX PYXON-20, Tokyo, Japan) and diluted 10-fold using sterile phosphate-buffered saline. The mixture was homogenized using a stomacher (Wisemix WES-400; DAIHAN Scientific, Korea) at level 10 for 1 min. The total viable count (TVC) and psychrophilic bacteria were counted on plate count agar (Merck, Darmstadt, Germany) and standard plate count agar (Oxoid Ltd., Hampshire, UK), respectively. Each agar was then incubated at 37°C for 48 h and at 25°C for 72 h. *Escherichia coli* (*E. coli*), coliforms, and lactic acid bacteria (LAB), were counted using Petrifilm (3M, St. Paul, MN, USA). TVC, *E. coli*, coliforms, and LAB were incubated at 37°C for 48 h. The microbial colonies (30 to 300) were counted and expressed as log_10_ colony forming unit (CFU)/g sample.

### Scanning electron microscopy

Cooked duck breast meat was cut into pieces with a dimension of 5×5×5 mm and freeze-dried at −40°C for 30 h. The freeze-dried samples were coated with platinum for 120 s. The section of the samples was scanned using a scanning electron microscope (Hitachi SU8010; HITACHI, Tokyo, Japan) at 3.0 kV, 8.8 mm×150 LM (UL) at 100× magnification.

### Statistical analysis

Data were expressed as means±standard deviation. Statistical analysis was processed using SPSS-PASW statistics software version 20.0 for Windows, with one-way analysis of variance. Tuckey’s post hoc test (p<0.05) was used to define the difference among the treatments.

## RESULTS AND DISCUSSION

### Cooking loss and color of the duck breast meat

The results of the physicochemical and color analyses are shown in Table 1. Cooking loss in breast meat cooked using the sous-vide method ranged from 5.20% to 39.26%. Cooking loss in the samples cooked at 80°C for 180 min was significantly higher than that in the samples cooked at other temperatures for 60 or 180 min (p<0.05). The L*, b*, H°, and whiteness values increased, while the a* and C* values decreased with the increase in cooking time (Table 1). In this study, the cooking loss in the duck breast meat samples was directly proportional to the cooking temperature and time (p<0.05). Cooking loss is closely associated with lightness, shear force, and texture properties [12]. Decreasing cooking loss has economic benefits with respect to the meat industry as it increases the yield of the final product [13]. Approximately 80% of water in the muscle is trapped within the myofibrils [14]. The muscle fibers shrink and aggregate during the cooking process, which is attributed to the heat-induced muscle fiber damage. The deformation of muscle fiber decreases the physical space retaining free water in the meat and increases cooking loss [12]. Previous studies have reported that cooking loss increases with an increase in cooking temperature [15]. Consistent with this finding, this study demonstrated that cooking loss was high in meat cooked at high temperature for a prolonged time. Color is one of the most important quality attributes that affects consumer acceptance [16]. Duck meat is considered red meat due to its abundance of red muscle fibers [7]. The heat-induced denaturation of myoglobin can affect the color of red meat [17]. In the sous-vide method, the meat is processed at low temperatures, which prevents complete myoglobin denaturation. Hence, meat cooked using the sous-vide method maintains its red color. Additionally, the sous-vide method involves prolonged cooking time, which enables uniform cooking of the meat [16]. The high values of L* and whiteness, which are associated with light scattering, result from the aggregation and denaturation of myofibrillar proteins [15]. The a* value indicates the degree of myoglobin denaturation [18]. Myoglobin is a structural protein that also determines the color of the meat. Myoglobin begins to undergo denaturation at 60°C. The degree of myoglobin denaturation determines the redness of the cooked meat [19]. In this study, cooking at high temperatures for prolonged time decreased the a* values but increased the b* values of the cooked meat. The a* values may decrease due to protein denaturation, which promotes brown coloration and met-myoglobin formation [15]. The C* values, which measure brightness, ranged from 14.49 to 22.55 at the tested cooking temperatures. The C* values decreased with the increase in cooking temperature. At constant cooking temperature, the C* value decreased with the increase in cooking time. The H° value of cooked breast was the highest in the samples cooked at 80°C for 180 min (p<0.05). These results are consistent with previous studies, which reported that the H° value of the cooked products increases with the increase in cooking temperature and time [18,19].

### Thiobarbituric acid reactive substance and volatile basic nitrogen values of the duck breast meat

**Figure 1:**
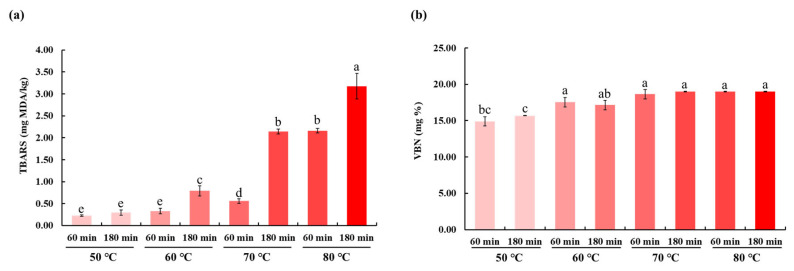
Evaluation of thiobarbituric acid reactive substance (TBARS) (a) and volatile basic nitrogen (VBN) (b) in the duck breast meat. The samples were cooked using the sous-vide method for 60 and 180 min at different temperatures (50°C, 60°C, 70°C, and 80°C). ^a–e^ Means with different letters are significantly different (p<0.05); values represent mean±standard deviation (n = 3).

The thiobarbituric acid reactive substance (TBARS) values are depicted in Figure 1. The TBARS values, which indicate the levels of lipid oxidation products, increased with the increase in cooking temperature at constant cooking time (p< 0.05). Similarly, the TBARS values increased with the increase in cooking time at a constant cooking temperature. Thus, the duck breast meat cooked using the sous-vide method at 80°C for 180 min exhibited the highest TBARS value. Lipid oxidation, which results from a complex free radical chain reaction, affects the rancidity of meat during storage [19]. The TBARS values of the duck breast reported in this study were higher than those reported for other meats, such as porcine or bovine. The TBARS value is dependent on the fatty acid composition of the duck fat [8]. The duck meat is rich in polyunsaturated fatty acid [20]. Although polyunsaturated fatty acid has various health benefits, it is susceptible to heat-induced oxidation [21]. In the sous-vide heating method, the duck meat is cooked at low temperatures (50°C to 60°C), which decreases the development of rancidity. This is consistent with the results of a previous study, which demonstrated that sous-vide cooking with low temperature could delay the increase in TBARS values [18].

The VBN content in the duck meat ranged from 14.89 to 19 mg% (Figure 1). The VBN content was not significantly different between the meat samples cooked at 60°C, 70°C, and 80°C, at both cooking times of 60 and 180 min. In contrast, the VBN contents in the duck breast meat cooked at 50°C for 60 or 180 min were markedly lower than those in the duck breast meat cooked at 60°C, 70°C, or 80°C. The VBN content is an indicator of protein deterioration in the meat [22]. VBN is one of the most important parameters to evaluate meat freshness [23]. The formation of volatile compounds is also influenced by cooking temperature and time [24]. Meat with a VBN value below 5 mg% is considered fresh, while values above 30 mg% indicate decomposition [25]. In this study, the VBN values of all the groups were within the acceptable range (14.89 to 19 mg%). This indicated that the sous-vide method does not promote protein deterioration in the duck meat.

### Microbiological properties of the duck breast meat

The microbial counts of duck breast are shown in Table 2. The counts of TVC, *E. coli*, coliform, and LAB in the raw meat were 4.11, 3.95, 3.98, and 2.14 log CFU/g, respectively. No growth of psychrotrophic bacteria was observed in the raw meat. The microbial counts of all cooked samples, except for the sample cooked at 50°C, were below the detection limit (2 log CFU/g). The counts of total viable bacteria, *E. coli*, and coliform in the samples cooked at 50°C for 60 min were 3.89, 3.61, and 3.63 log CFU/g, respectively. Only the total viable bacteria (2.92 log CFU/g) were detected in the samples cooked at 50°C for 180 min. The cooking temperature and time can affect the microbial profile of the meat [15]. Cooking temperature can inhibit the growth of bacteria in the meat, especially when the heat energy is delivered to the meat core. Sous-vide cooking method effectively inhibits bacterial growth as the meat is uniformly cooked. The analysis of the cut surface of sous-vide-cooked meat indicated uniform heat transfer, which can affect bacterial growth. In this study, the sous-vide cooking method effectively inhibited the growth of bacteria [26].

### The texture properties of the duck breast meat

The results of WBSF and TPA are shown in Table 3. Meat tenderness, which is measured based on the WBSF, is an important quality characteristic that influences consumer acceptance of meat products. The TPA is a crucial quality attribute of processed foods, as it affects their quality and palatability. The WBSF values, which indicate the force required to cut the meat, increased with longer cooking time and higher temperature. The WBSF value ranged from 1.89 to 6.96 kg, and different cooking conditions significantly affected the WBSF values of duck meat samples (p<0.05). Compared to the meat samples cooked at 50°C, the hardness was significantly higher in the meat samples cooked at 60°C, 70°C, or 80°C. Cooking time affected the hardness of the duck breast meat cooked at 50°C, and the duck breast meat cooked at 50°C for 180 min exhibited increased harness. The springiness, cohesiveness, chewiness and gumminess of the duck breast meat increased upon increasing the cooking temperature and time. The increase in cooking time and temperature resulting in increased WBSF and hardness may be attributed to the denaturation and aggregation of various proteins in duck meat. Most sarcoplasmic proteins aggregate between 40°C and 60°C, and heat aggregation of these proteins could extend up to 90°C [27]. Interestingly, when beef muscles are heated at low temperatures for a long time, sarcoplasmic proteins can undergo tenderization by some of enzymes. When myosin is hated above 65°C, some of the hydrophobic residues participate in protein─protein interactions to form gels for aggregation, while collagen and actin form aggregates between 65°C to 77°C, and between 80°C to 83°C, respectively [27]. Furthermore, heat treatment can cause myofibrils to shrink and water to be exuded from the muscle fibers. WBSF and texture profile are closely related to cooking loss, as meat texture is influenced by the content of immobilized water in the muscle fibers [28]. Preventing the denaturation of myofibrillar proteins can result in a softer texture and promote the retention of free water within the muscle fiber. Thus, the tenderness of sous-vide cooked duck breast can be influenced by factors such as water holding capacity, protein denaturation and aggregation, which depend on the specific cooking conditions.

### Microstructure of the duck breast meat

The microstructure of duck breast meat cooked using the sous-vide method is shown in Table 4. The myofibrils showed increased contraction with higher cooking temperatures and longer cooking times. Moreover, increasing the cooking temperature and time resulted in decreased redness of the samples and increased coarseness of the meat surface. Scanning electron microscopy (SEM) analysis showed that myofibril contraction and meat density increased with higher cooking temperature, while keeping the cooking time constant. In addition, increasing the cooking time, while keeping the cooking temperature constant, resulted in increased meat density. The pore size of the duck breast meat decreased with higher cooking temperatures and longer cooking times. The morphology of the duck breast meat exhibited consistency with its microstructure at different cooking temperatures and cooking times. The muscle tissue of the animal undergoes shrinkage and contraction upon heating, which is attributed to myofibrillar protein denaturation. The sarcomere length of the muscle fiber progressively decreases with higher cooking temperature [29]. Sarcomeres and myofibrils start shrinking at temperature between 40°C and 50°C, while the connective tissue and muscle fibers typically contract longitudinally at temperature between 60°C and 70°C [15]. Moreover, heating causes denaturation of myoglobin in the red meat, resulting in the loss of red color. The findings of this study revealed that the duck breast meat cooked using the sous-vide method showed myofibrillar shrinkage and discoloration mediated by myoglobin denaturation. SEM analysis showed that the meat structure density increased with higher cooking temperatures and longer cooking time, which can be attributed to the contraction of myofibrillar and connective tissues. This may explain the tenderness of the samples cooked at low temperatures.

## CONCLUSION

This study evaluated the effect of sous-vide cooking temperature and time on cooking loss, color, texture, lipid oxidation, protein deterioration, and microstructure of the duck breast meat. The results of our study showed that cooking duck breast meat using sous-vide at a temperature of 60°C for at least 60 min resulted in acceptable tenderness and minimized rancidity. However, samples cooked at 50°C showed contamination with *E. coli* and coliforms. As a result, duck breast meat might be cooked at temperatures higher than 60°C for at least 60 min. The sous-vide cooking method helps maintain the quality attributes of the cooked duck breast meat, including texture and microbial safety. Additional studies are warranted to assess the effect of storage on the quality characteristics and sensory profiles of the duck breast cooked using the sous-vide method.

## Figures and Tables

**Table 1:** Physicochemical parameters of duck breast meat cooked using the sous-vide method

Temperature (°C)	Time (min)	Cooking loss (%)	L*^1)^	a*	b*	Chroma	Hue°	Whiteness
50	60	5.20±0.38^e^	57.10±0.70^e^	19.78±0.77^a^	10.15±0.76^c^	22.55±0.90^a^	27.60±1.70^e^	51.82±0.78^e^
	180	8.79±0.27^de^	64.33±1.08^c^	16.78±0.53^b^	9.71±0.37^c^	19.87±0.59^bc^	31.96±1.22^d^	59.18±0.45^c^
60	60	13.61±1.02^d^	61.41±0.86^d^	19.29±0.95^a^	11.08±0.30^b^	23.09±1.49^a^	30.34±0.72^d^	55.60±1.02^d^
	180	25.93±1.01^b^	65.44±0.76^b^	13.63±0.79^d^	11.07±0.53^b^	17.77±0.71^d^	40.27±1.31^c^	61.38±0.74^b^
70	60	19.91±1.47^c^	67.46±1.39^b^	15.64±1.21^bc^	12.65±0.49^a^	20.88±0.87^b^	38.65±1.77^c^	61.88±1.44^b^
	180	28.83±1.15^b^	66.83±0.65^a^	14.78±1.31^cd^	11.35±0.62^b^	18.52±0.89^cd^	38.83±0.66^c^	61.73±1.08^ab^
80	60	35.89±2.90^a^	66.63±0.82^a^	9.40±0.81^e^	12.30±0.56^a^	15.48±0.78^e^	53.19±2.13^b^	63.54±0.66^a^
	180	39.26±8.40^a^	65.43±1.14^a^	7.56±0.35^f^	12.71±0.48^a^	14.49±0.67^e^	57.49±1.03^a^	62.49±1.66^ab^

1) L*, lightness; a*, redness; b*, yellowness; Hue°, Hue angle.

a–e Means within a column with different letters differ significantly (p<0.05).

**Table 2:** Microbial profile of duck breast meat cooked using the sous-vide method

Temperature (°C)	Time (min)	Microorganism (log10 CFU/g)				

		TVB	*E. coli*	Coliform	LAB	Psychrophilic
Raw meat	0	4.11±0.07^a^	3.95±0.02^a^	3.98±0.02^a^	2.14±0.09	N/D
50	60	3.89±0.04^b^	3.61±0.01^b^	3.63±0.06^b^	N/D	N/D
	180	2.92±0.09^c^	N/D	N/D	N/D	N/D
60	60	N/D	N/D	N/D	N/D	N/D
	180	N/D	N/D	N/D	N/D	N/D
70	60	N/D	N/D	N/D	N/D	N/D
	180	N/D	N/D	N/D	N/D	N/D
80	60	N/D	N/D	N/D	N/D	N/D
	180	N/D	N/D	N/D	N/D	N/D

CFU, colony forming unit; TVB, total viable bacteria; *E. coli*, *Escherichia coli*; LAB, lactic acid bacteria.

a–c Means within a column with different letters differ significantly (p<0.05).

**Table 3:** Texture properties of duck breast meat cooked using the sous-vide method

Temperature (°C)	Time (min)	WBSF (kg)	Hardness (kg)	Springiness	Cohesiveness	Chewiness (kg mm)	Gumminess (kg)
50	60	1.89±0.08^c^	0.52±0.07^c^	0.56±0.06^c^	0.39±0.04^c^	0.13±0.03^d^	0.25±0.03^e^
	180	2.47±0.18^c^	0.77±0.15^b^	0.57±0.03^c^	0.41±0.03^ab^	0.17±0.02^d^	0.28±0.03^e^
60	60	2.76±0.60^c^	1.13±0.13^a^	0.58±0.05^c^	0.41±0.05^ab^	0.25±0.03^c^	0.43±0.04^d^
	180	4.16±0.27^bc^	1.19±0.18^a^	0.60±0.05^c^	0.45±0.05^ab^	0.29±0.05^c^	0.47±0.06^cd^
70	60	4.57±0.84^bc^	1.25±0.97^a^	0.62±0.03^bc^	0.45±0.03^ab^	0.35±0.03^bc^	0.58±0.03^ab^
	180	5.19±0.27^b^	1.20±0.85^a^	0.67±0.10^bc^	0.46±0.10^ab^	0.38±0.05^a^	0.50±0.05^bc^
80	60	4.79±0.45^b^	1.22±0.65^a^	0.80±0.07^a^	0.53±0.07^a^	0.39±0.05^a^	0.56±0.03^a^
	180	6.96±0.67^a^	1.28±0.67^a^	0.72±0.10^ab^	0.46±0.10^b^	0.35±0.03^ab^	0.49±0.02^c^

WBSF, Warner-Bratzler shear force.

a–e Means within a column with different letters differ significantly (p<0.05).

**Table 4:** Images and microstructures of duck breast meat cooked using the sous-vide method

Time (min)	Type	Temperature (°C)			

		50	60	70	80
60	Image	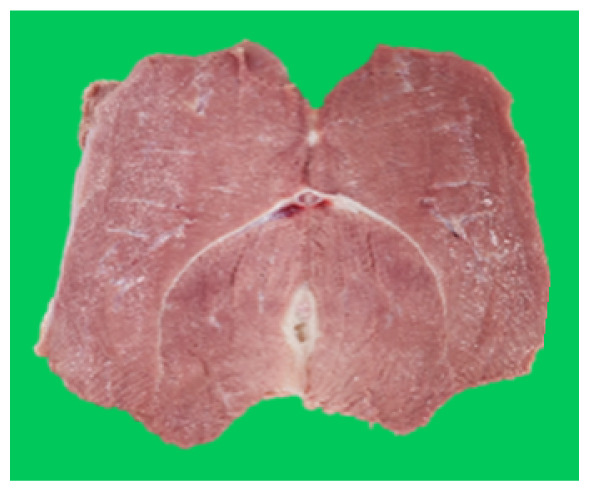	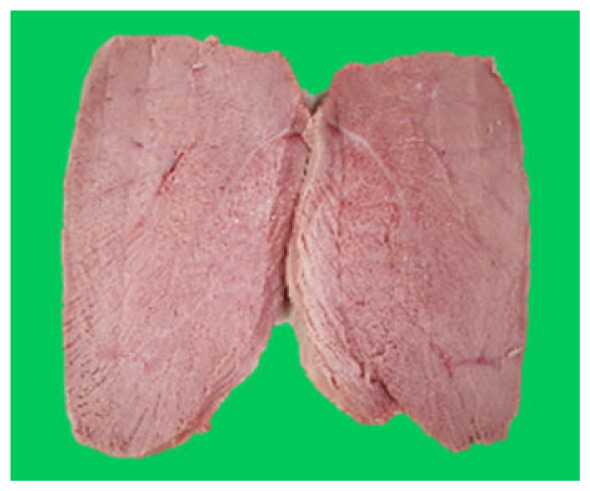	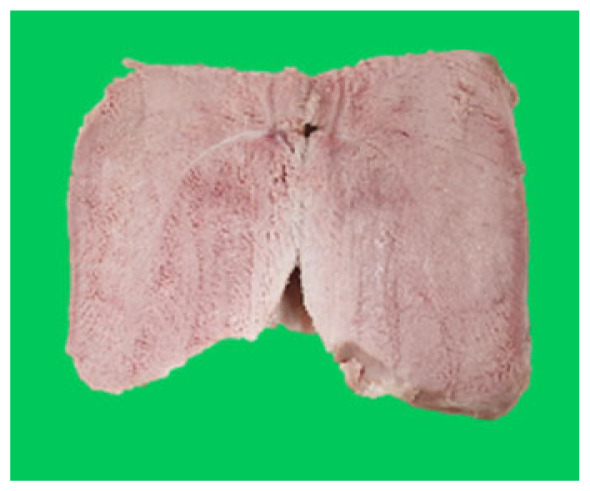	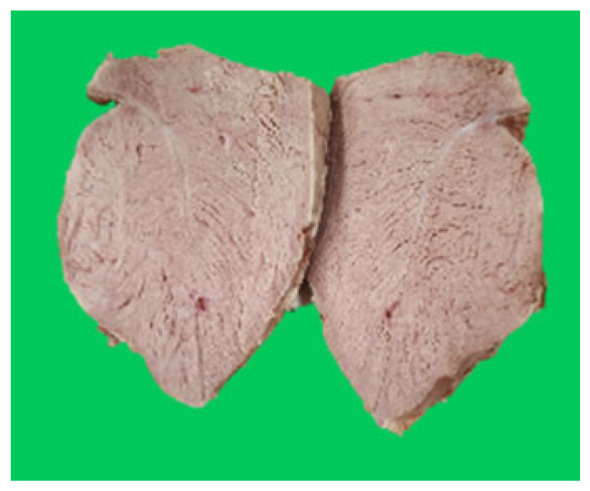
	SEM	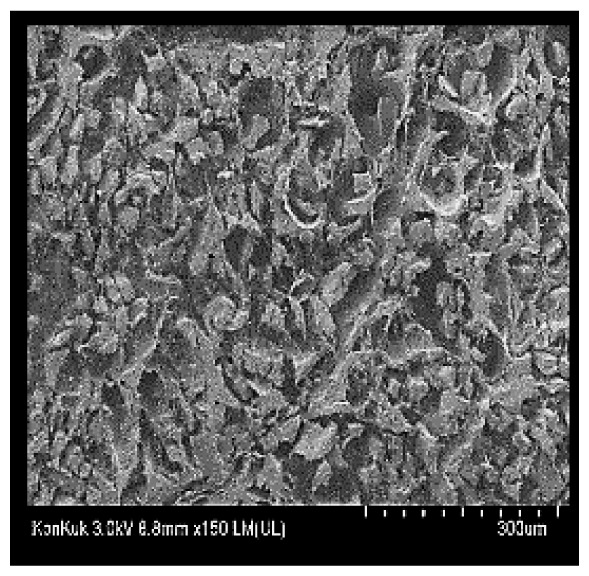	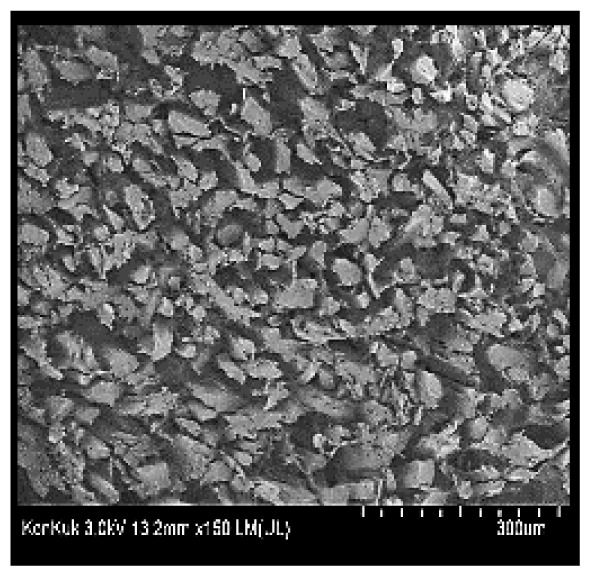	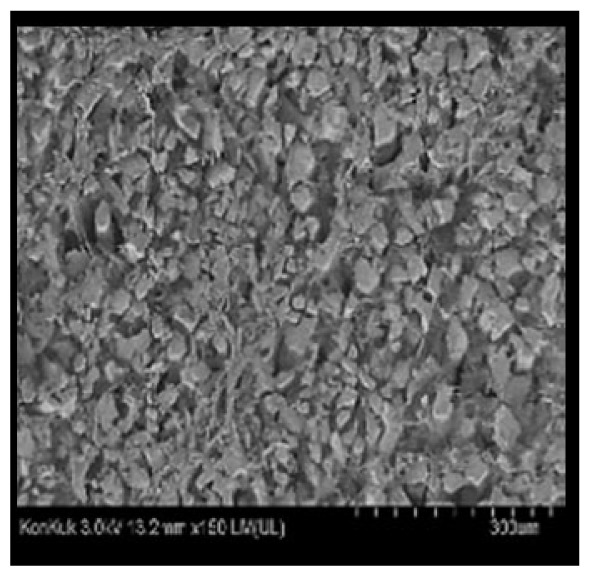	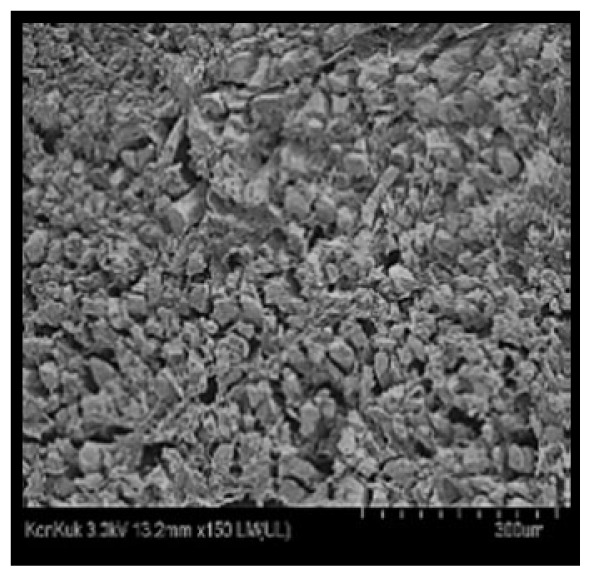
180	Image	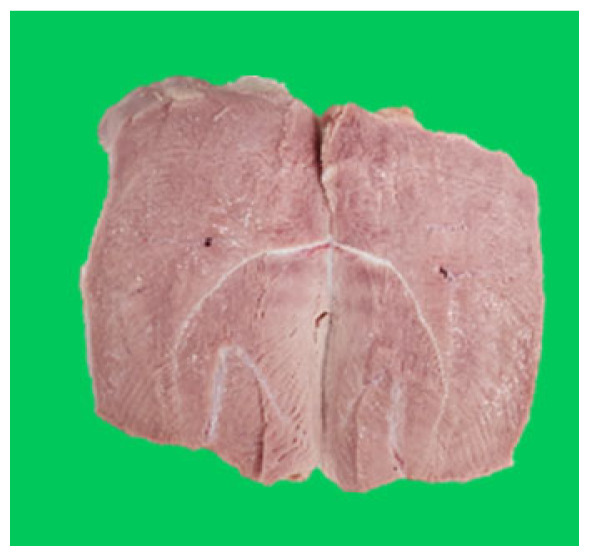	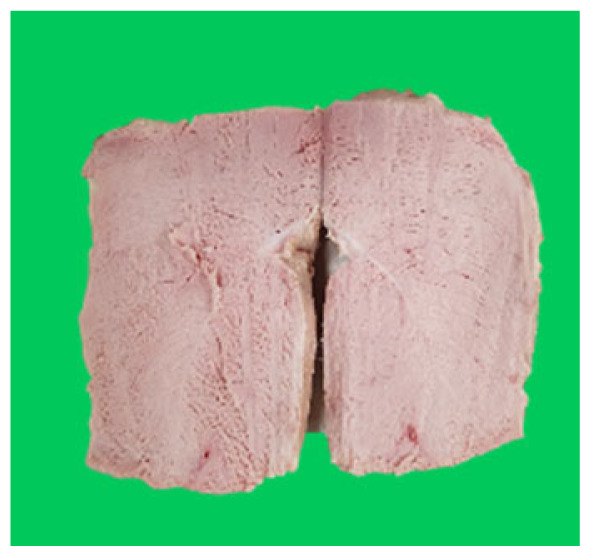	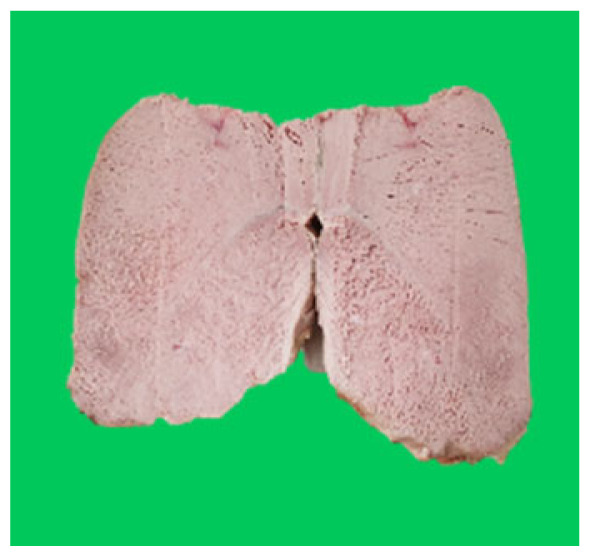	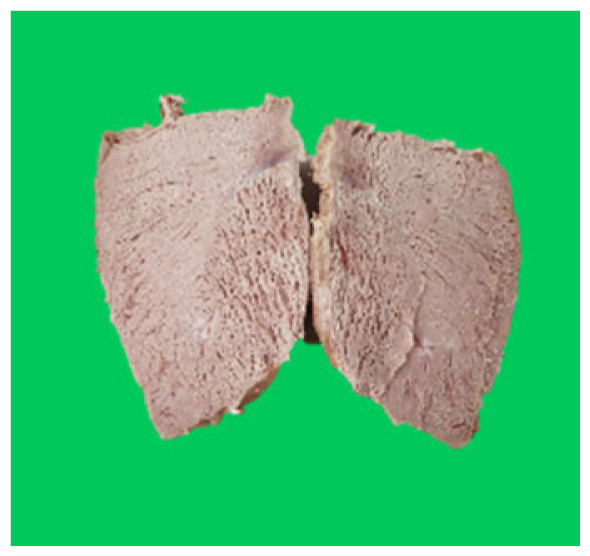
	SEM	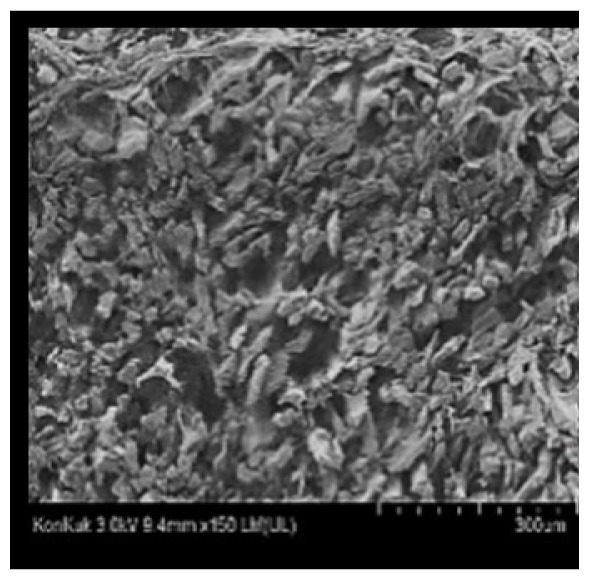	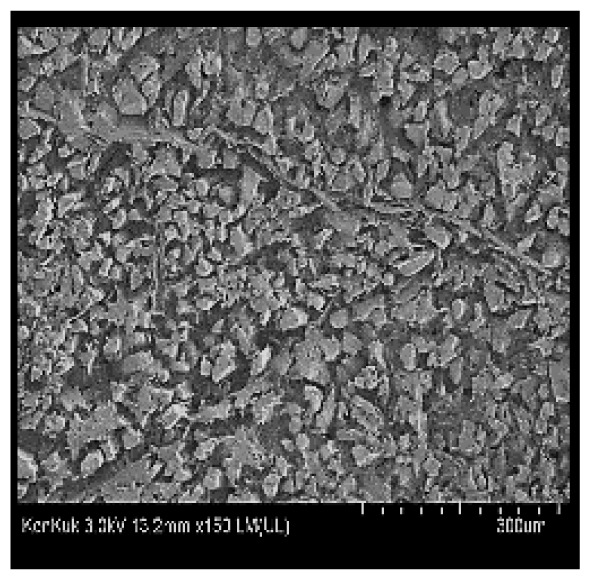	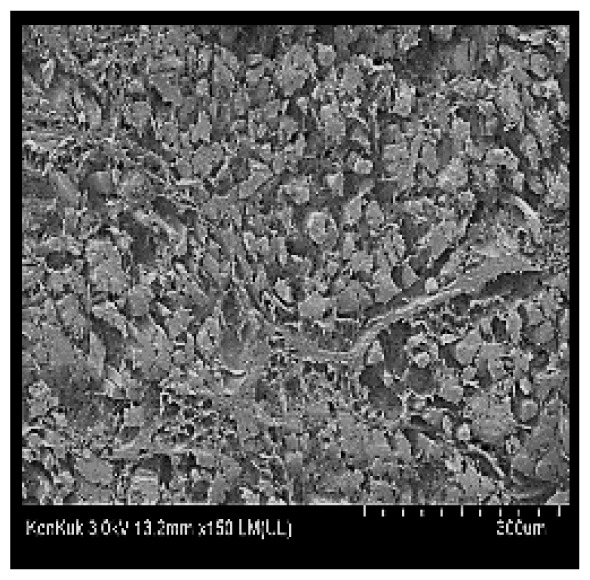	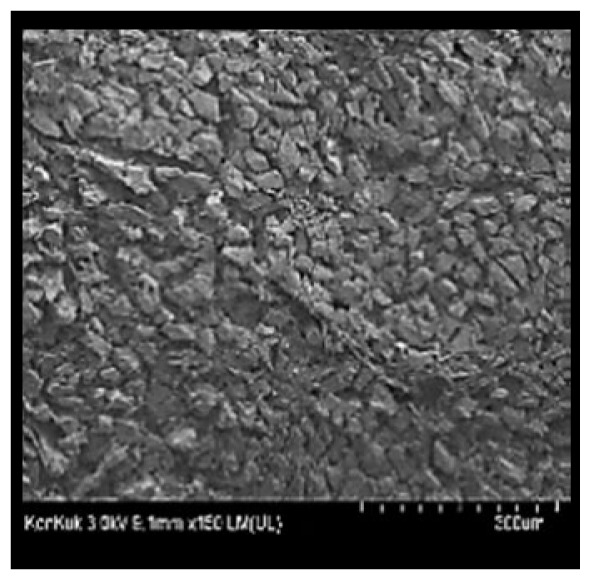

SEM, scanning electronic microscopy.
